# Targeted metabolomics analysis of postoperative delirium

**DOI:** 10.1038/s41598-020-80412-z

**Published:** 2021-01-15

**Authors:** Bridget A. Tripp, Simon T. Dillon, Min Yuan, John M. Asara, Sarinnapha M. Vasunilashorn, Tamara G. Fong, Eran D. Metzger, Sharon K. Inouye, Zhongcong Xie, Long H. Ngo, Edward R. Marcantonio, Towia A. Libermann, Hasan H. Otu

**Affiliations:** 1 Department of Electrical and Computer Engineering, University of Nebraska-Lincoln, Nebraska Hall E419, P.O. Box 880511, Lincoln, NE 68588 USA; 2 PhD Program of Complex Biosystems, University of Nebraska-Lincoln, Lincoln, USA; 3 Proteomics, Bioinformatics and Systems Biology Center, Beth Israel Deaconess Medical Center, Boston, USA; 4 Harvard Medical School, Boston, USA; 5 Division of Signal Transduction and Mass Spectrometry Core, Beth Israel Deaconess Medical Center, Boston, USA; 6 Department of Medicine, Beth Israel Deaconess Medical Center, Boston, USA; 7 Harvard T.H. Chan School of Public Health, Boston, USA; 8 Department of Neurology, Beth Israel Deaconess Medical Center, Boston, USA; 9 Aging Brain Center, Marcus Institute for Aging Research, Hebrew SeniorLife, Boston, USA; 10 Department of Medicine, Hebrew SeniorLife, Boston, USA; 11 Department of Psychiatry, Beth Israel Deaconess Medical Center, Boston, USA; 12 Department of Anesthesia, Critical Care and Pain Medicine, Massachusetts General Hospital, Boston, USA

**Keywords:** Cognitive ageing, Metabolomics, Bioinformatics, Metabolomics, Neurological disorders

## Abstract

Postoperative delirium is the most common complication among older adults undergoing major surgery. The pathophysiology of delirium is poorly understood, and no blood-based, predictive markers are available. We characterized the plasma metabolome of 52 delirium cases and 52 matched controls from the Successful Aging after Elective Surgery (SAGES) cohort (N = 560) of patients ≥ 70 years old without dementia undergoing scheduled major non-cardiac surgery. We applied targeted mass spectrometry with internal standards and pooled controls using a nested matched case-control study preoperatively (PREOP) and on postoperative day 2 (POD2) to identify potential delirium risk and disease markers. Univariate analyses identified 37 PREOP and 53 POD2 metabolites associated with delirium and multivariate analyses achieved significant separation between the two groups with an 11-metabolite prediction model at PREOP (AUC = 83.80%). Systems biology analysis using the metabolites with differential concentrations rendered “valine, leucine, and isoleucine biosynthesis” at PREOP and “citrate cycle” at POD2 as the most significantly enriched pathways (false discovery rate < 0.05). Perturbations in energy metabolism and amino acid synthesis pathways may be associated with postoperative delirium and suggest potential mechanisms for delirium pathogenesis. Our results could lead to the development of a metabolomic delirium predictor.

## Introduction

Delirium is a condition characterized by an acute change and fluctuation in attention, thinking, and consciousness. Postoperative delirium complicates the courses of 15–53% of older adults undergoing major surgical procedures^1,2^, and is associated with higher rates of postoperative complications^3^, longer hospital stays^4^, higher rates of discharge to extended care facilities^5^, and increased mortality^4–6^. The economic impact of delirium associated health care expenditures is over $164 billion per year in the U.S. alone^7^.

Currently, delirium is solely a clinical diagnosis, and there is no laboratory test or biomarker to facilitate its diagnosis. Additionally, delirium etiology and pathogenesis are poorly understood. Previous research has suggested several mechanisms for delirium pathogenesis, which include: (1) increased expression of inflammatory markers found in the blood and central nervous system^8–12^; (2) alterations in plasma levels of precursor amino acids leading to dysfunction in neurotransmitter systems^13^; (3) oxidative stress leading to cerebral dysfunction^14–16^; (4) acute or chronic stress response resulting in aberrantly high levels of glucocorticoids^17,18^; and (5) dysregulation of the circadian rhythm^15,19–21^.

Metabolomics is a high-throughput quantitative approach to study the metabolome—the collection of small metabolites found in a system. These smaller metabolic molecules are often the end products of the biochemical processes in a cell and are particularly sensitive to endogenous and exogenous stimuli^22^. Differences in their concentration levels provide an efficient way to monitor and detect alterations in specific cellular pathways. Metabolomics can reveal transient biochemical changes that are closely aligned with the disease state of a system^22^.

Metabolomics has been extensively used to characterize cellular changes in disease phenotypes and facilitate identification of disease-specific markers^23–25^. Targeted metabolomics approaches have been employed previously in identifying novel biomarkers and distinguishing between diseased and healthy cohorts^26,27^. Only a few prior studies have explored metabolic changes associated with delirium and illuminating potential underlying biological mechanisms^28–31^.

To obtain more insights into the pathophysiological mechanisms of delirium as well as to identify potential risk and disease markers, we performed targeted metabolomic profiling both at preoperative and postoperative time points. We identified multiple metabolites linked to delirium before and after surgery and demonstrated enrichment of several metabolic pathways. Our associative, predictive, and systems analysis may help to identify pathophysiologic pathways to prioritize for development of diagnostic and therapeutic regimens for postoperative delirium.

## Results

**Figure 1:**
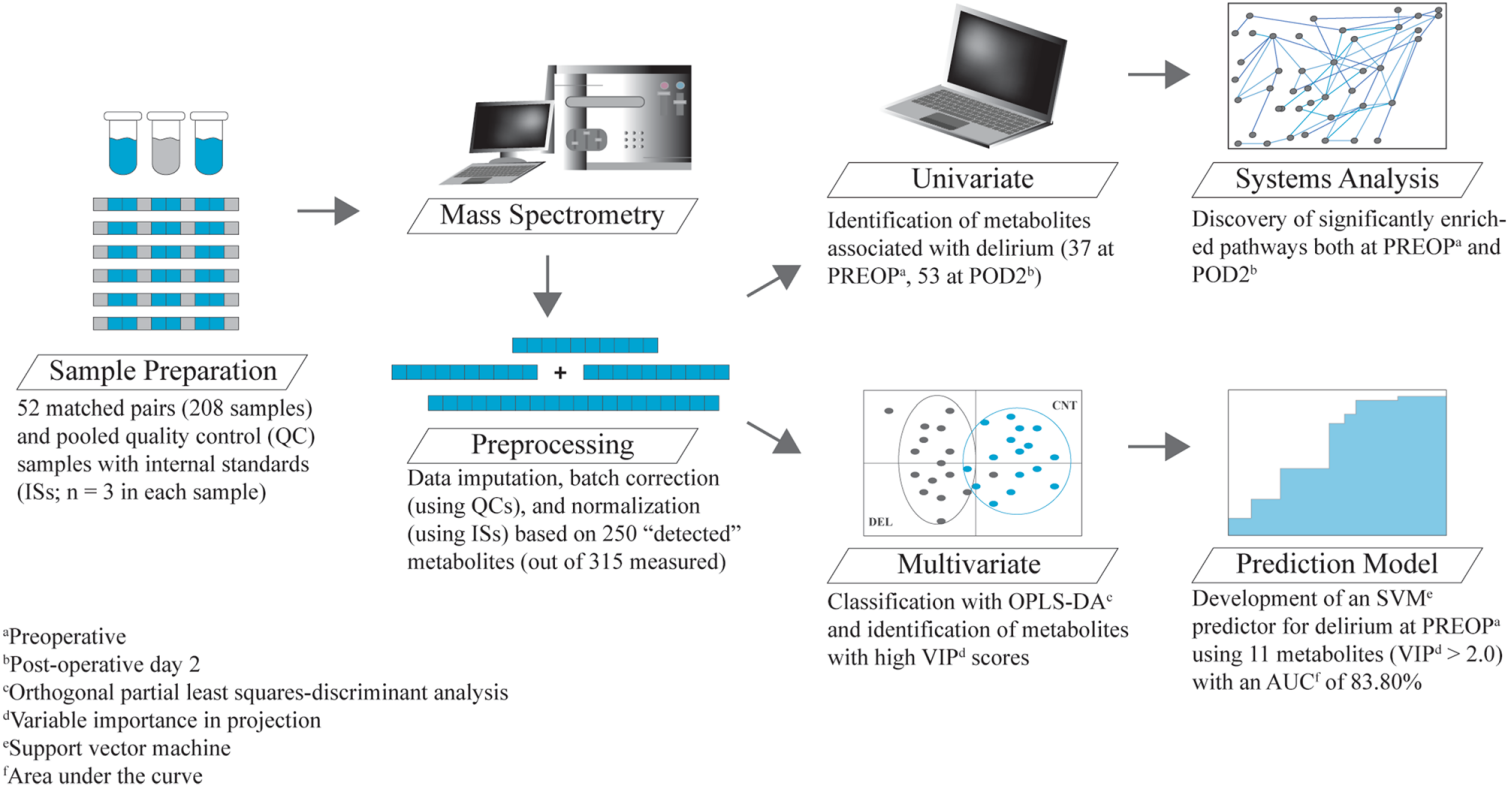
Experimental design workflow. Key steps in our experimental design included: sample preparation, mass spectrometric qualitative and quantitative analysis, data preprocessing, univariate and multivariate statistical analysis, systems analysis applied to the univariate findings, and predictive modeling using multivariate results.

In Fig. 1, we show the overall workflow of the experimental and computational steps applied in this paper.

### Sample characteristics

Table 1 shows the sample demographics and clinical variables of the metabolomics patient samples. On average, patients in the matched sample (n = 104; 52 matched pairs) were 77 years old, 53% female, and 49% had a vascular comorbidity. The characteristics of the delirium cases (DEL) and no-delirium controls (CNT) were similar, indicating a successful match procedure. In terms of non-match variables, on average both cases and control were overweight and showed little evidence of malnutrition. Both cases and controls reported poor sleep in the hospital, which is typical^32^, with cases experiencing slightly more sleep disruption. The prevalence of diabetes medication use was low and similar in cases and controls. We did not control for the variables that were not used in the matching process because of the risk of over-controlling for variables that are potentially along the causal pathway to delirium^33,34^.

**Table 1:** Sample characteristics in the matched cohorts.

Study variables	Full sample	Delirium	No delirium
	(N = 104)	(N = 52)	(N = 52)
Age (M, SD)^1^	77.1 (4.7)	77.3 (5.0)	77.0 (4.4)
Female (%)^1^	56 (53%)	28 (55%)	28 (55%)
Nonwhite, n (%)	10 (9%)	5 (9%)	5 (9%)
Education in years (M, SD)	15.0 (2.8)	15.0 (3.0)	15.1 (2.7)
GCP (M, SD)^1^	55.6 (5.4)	55.2 (5.5)	56.0 (5.4)
Charlson score (M, SD)	1.3 (1.4)	1.3 (1.4)	1.3 (1.5)
Vascular comorbidity, n (%)^1^	52 (49%)	26 (49%)	26 (49%)
ApoE ɛ4 carrier, n (%)^1^	24 (23%)	12 (23%)	12 (24%)
**Anesthesia type, n (%)**			
General	85 (83%)	42 (82%)	43 (84%)
Spinal	17 (17%)	9 (18%)	8 (16%)
Both	0 (0%)	0 (0%)	0 (0%)
PREOP body mass index (M, SD)	29.0 (4.9)	29.9 (5.5)	28.2 (4.1)
Unintentional weight loss in past year (≥ 10 lbs)^2^	7 (8%)	2 (5%)	5 (11%)
PREOP albumin (g/dL, M, SD)^3^	4.3 (0.4)	4.4 (0.4)	4.2 (0.4)
Any problems with sleep in the past night, n (%)^4^	83 (83%)	44 (92%)	39 (75%)
In-hospital sleep problem is new or worse, n (%)^5^	72 (88%)	39 (91%)	33 (85%)
Taking diabetes medication on admission	17 (16%)	8 (15%)	9 (17%)

^1^Variable used for matching.

^2^Full sample N = 85 (delirium n = 39, no delirium n = 46).

^3^Full sample N = 34 (delirium n = 15, no delirium n = 19); albumin was tested only when malnutrition was suspected.

^4^Positive response to question on either POD1 or POD2; Sample for this question: N = 100 (delirium n = 48, no delirium n = 52).

^5^Sample for this question: N = 82 (delirium n = 43, No delirium n = 39).

*Abbreviations*: ApoE  =  Apolipoprotein E, GCP  =  general cognitive performance, M  =  mean, 3MS  =  Modified Mini-Mental State Examination, SD  =  standard deviation, PREOP  =  preoperative, POD  =  postoperative day.

### Data pre-processing

We identified 250 metabolites as “present,” i.e., detected at a level suitable for downstream analysis (out of 315 metabolites measured). Missing data accounted for 3.15% of total data acquired from present metabolites (Supplementary Table S1), which was much smaller than the typical amount of missing data percentage seen in metabolomics studies (15–26%)^35–39^. After data imputation, signal drift correction, and normalization, the percent relative standard deviation (RSD) was less than 5 for 87% and less than 10 for 98% of all metabolites in the pooled QC samples (Supplementary Tables S2, S3, S4).

### Metabolites altered in delirium group at PREOP

**Figure 2:**
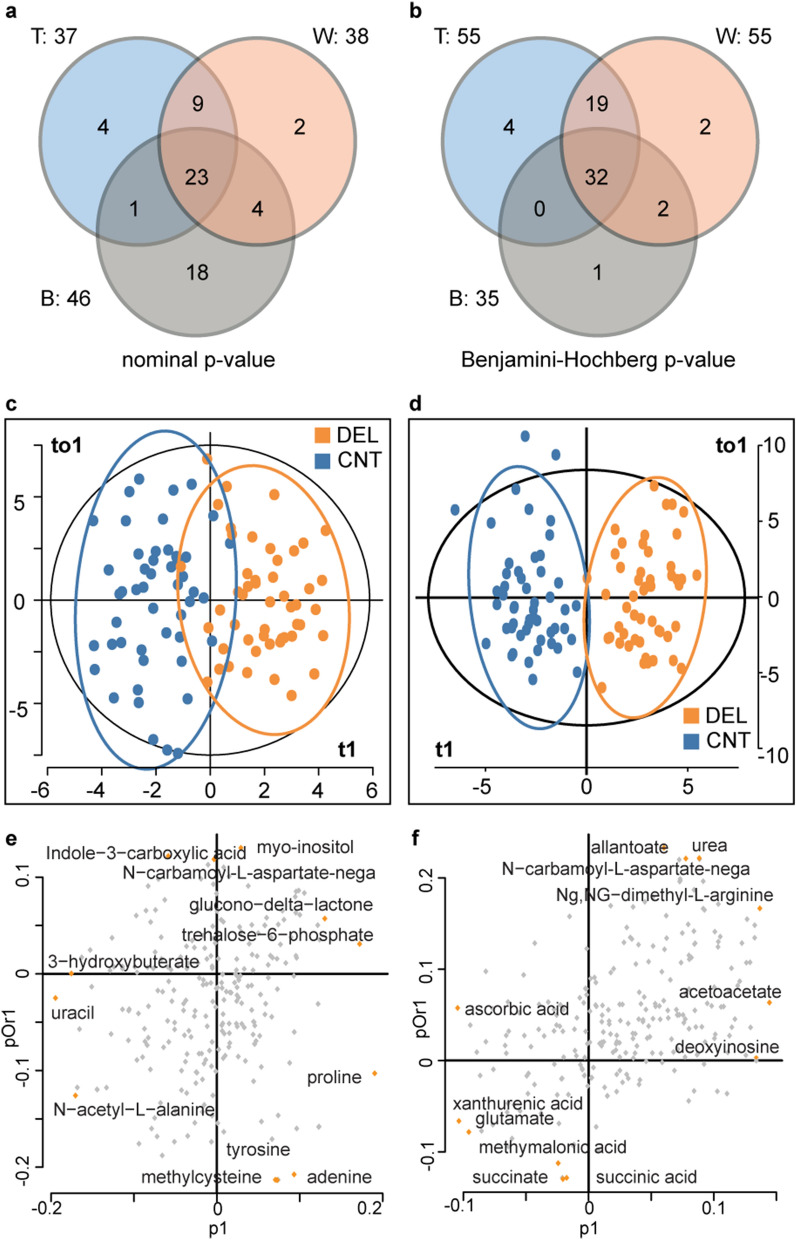
Statistical approaches to discovering significant metabolites at PREOP and POD2. For (**a**, **b**), we applied both parametric [t-test (T)] and nonparametric [Wilcoxon Rank (W) and binomial (B)] statistical tests to account for the degree, direction, and rank of difference between delirium (DEL) and control (CNT) groups at both preoperatively (PREOP) and post-operative day 2 (POD2) time points. To correct for multiple hypothesis testing, we used the Benjamini–Hochberg (BH) procedure. A metabolite was considered to have differentially quantified concentrations if it had a BH-corrected p value < 0.05 in at least two statistical tests. (**a**) At PREOP, none of the metabolites met our strict criteria for differential concentration. Four metabolites had a BH-corrected p value < 0.05 only in the binomial test (see text). Systems biology was performed using the 37 metabolites that passed two or more tests with a nominal p value < 0.05. (**b**) At POD2, there were 53 metabolites that met our criteria for differential concentration. These metabolites were used as input for systems analysis. Score plots for the OPLS-DA analysis using the (**c**) PREOP and (**d**) POD2 data. Ellipses represent clustering based on the Mahalanobis distance for outlier detection (orange: delirium, blue: control, and black: all samples). Metabolites with the most extreme loadings (positive and negative) for (**e**) PREOP and (**f**) POD2 are noted. These metabolites had the greatest impact on the model.

Thirty-seven metabolites were found to be altered in DEL at PREOP (nominal *p* value < 0.05 in at least two tests) when compared with CNT (Fig. 2a; Table 2a; Supplementary Table S5). Four of these metabolites had a binomial test BH-corrected *p* value < 0.05: trehalose-6-phosphate, phenyllactic acid, creatine, and N-acetyl-l-alanine. We performed 10,000 tenfold splits where at each iteration 1/10th of the samples were left out in each group, and the three statistical tests were run on the remaining samples. Twenty-eight of the thirty-seven metabolites had a nominal *p* value < 0.05 in at least two tests more than 50% of the time, thus showing high robustness (Supplementary Table S6).

**Table 2:** Metabolites with significant differential concentrations between delirium and control groups.

**a** Preoperative (PREOP) Metabolites	VIP	AVGp	FC	Metabolites	VIP	AVGp	FC
Trehalose-6-phosphate^3^	2.51	7.6E−04	1.52	Proline^3^	2.78	0.02	1.26
Phenyllactic acid	1.94	1.1E−03	− 1.19	N-acetyl-glutamate^3^	2.20	0.02	− 1.03
Creatine^3^	2.46	1.2E−03	− 1.36	Homocysteic acid	1.40	0.02	1.00
N-Acetyl-l-alanine^3^	2.49	1.3E−03	− 1.37	Dimethylglycine	1.86	0.02	− 1.22
S-Methyl-5-thioadenosine^1,2^	1.56	3.5E−03	− 1.47	Uridine^3^	2.42	0.02	− 1.10
Uracil^3^	2.84	4.2E−03	− 1.32	d-glyceraldehdye-3-phosphate	1.23	0.02	− 1.22
2-Oxobutanoate^1,2,3^	2.38	4.4E−03	− 1.08	Cytidine^1^	1.30	0.02	0.77
5-Methyl-THF	1.85	0.01	− 1.16	Glycolate	1.54	0.02	− 1.33
3-Hydroxybuterate^3^	2.56	0.01	− 1.69	N-Acetyl-glucosamine^1^	1.40	0.03	1.34
Allantoin	1.78	0.01	1.01	dl-Pipecolic acid	1.12	0.03	− 0.94
Citrulline	1.54	0.01	0.86	l-Isoleucine_15N	1.37	0.03	− 1.25
Creatinine^3^	2.11	0.01	− 1.02	d-gluconate	1.48	0.03	0.92
dTDP	0.58	0.01	− 1.37	Folate	1.31	0.03	− 1.05
Pyridoxine	1.41	0.01	− 1.19	Xanthine	1.44	0.04	− 0.68
S-Ribosyl-l-homocysteine^1,2^	1.66	0.01	− 0.86	Isoleucine	1.15	0.04	− 1.12
Glucono-delta-lactone^1^	1.90	0.01	1.19	Nicotinamide ribotide	0.95	0.05	− 0.83
FAD	1.78	0.01	1.04	Nicotinamide^3^	2.11	0.05	− 0.66
Valine	1.05	0.02	− 1.30	Nicotinamide Riboside	1.08	0.07	0.74
Dehydroascorbic acid	1.88	0.02	− 1.02				

**b** Postoperative Day 2 (POD2) Metabolites	VIP	AVGp	FC	Metabolites	VIP	AVGp	FC
p-Hydroxybenzoate	1.96	1.1E−04	2.05	Aconitate	1.33	0.02	1.43
Citrate	2.02	2.5E−04	1.25	Kynurenic acid	1.34	0.03	1.45
Acetoacetate	2.25	7.5E−04	1.54	S-Ribosyl-l-homocysteine^1^	1.28	0.03	1.11
Citraconic acid/itaconic acid^4^	2.01	1.0E−03	1.38	Ascorbic acid	1.63	0.03	− 0.94
Itaconic acid	1.96	1.0E−03	1.37	Maleic acid	1.44	0.03	1.36
2-Ketohexanoic acid	1.65	1.2E−03	1.46	Orotate	1.21	0.03	1.37
Deoxyinosine	2.09	1.4E−03	1.90	Atrolactic acid	1.53	0.03	1.28
N-acetyl-glucosamine	2.07	1.6E−03	1.41	Fumarate	1.41	0.03	1.30
Myo-inositol	1.97	2.0E−03	1.27	Xanthurenic acid	1.61	0.03	− 1.21
Isocitrate	1.91	2.1E−03	1.52	2-Keto-isovalerate	1.36	0.03	1.36
5-Phosphoribosyl-1-pyrophosphate	1.68	2.5E−03	1.45	Glutathione disulfide	1.75	0.03	1.28
dAMP	1.73	4.0E−03	1.56	Glycerophospho-choline	1.00	0.03	− 1.50
2-Hydroxy-2-methylbutanedioic acid	1.78	4.5E−03	1.38	d-Glucono-delta-lactone-6-phosphate	1.75	0.03	0.81
3-S-Methylthiopropionate	1.43	4.6E−03	1.82	S-Methyl-5-thioadenosine	1.19	0.03	1.00
Ng,NG-dimethyl-l-arginine	2.13	0.01	1.10	SBP	1.44	0.03	1.10
Citrate-isocitrate	1.57	0.01	1.23	S-Adenosyl-l-homocysteine	1.86	0.03	1.02
Cytidine^1^	2.07	0.01	1.57	d-Sedoheptulose-1-7-phosphate	1.31	0.03	1.06
1-Methyladenosine	1.88	0.01	1.55	Glucono-delta-lactone	1.43	0.03	0.74
GTP	1.82	0.01	1.56	Acetylphosphate	1.48	0.04	1.23
d-Gluconate	1.64	0.01	0.99	α-Ketoglutarate	1.72	0.04	1.26
4-Phosphopantothenate	1.57	0.01	1.60	Oxaloacetate	1.09	0.04	1.43
Dihydroorotate	1.32	0.01	1.22	2-Oxobutanoate	1.02	0.04	1.10
Glyoxylate	1.25	0.02	1.45	1-Methyl-Histidine	1.38	0.04	0.97
O8P-O1P	1.61	0.02	1.28	Homocysteine	1.78	0.04	1.66
d-Glucarate^1^	1.62	0.02	1.11	dUMP	1.11	0.04	0.72
Acetyllysine	1.31	0.02	1.54	Glucosamine	1.58	0.04	1.57
S-Adenosyl-l-methionine	1.62	0.02	1.48				

Metabolites were considered significant if they passed two or more statistical tests. The AVG-p is the average of the *p* values that passed cutoff. Variable importance in projection (VIP) score was obtained from orthogonal partial least squares discriminant analysis (OPLS-DA). VIPs reflect both the loading weights and the variability of each response. Fold-change (FC) of each metabolite was calculated by applying the one-step Tukey’s biweight algorithm on traditionally calculated FC values (Delirium/Control) for each paired sample. A negative FC denotes downregulation in the delirium group. **a)** PREOP: 37 metabolites statistically significant with a nominal *p* value < 0.05. **b)** POD2: 52 metabolites statistically significant with a BH-corrected *p* value < 0.05.

^1^Metabolites found to have significant differentially quantified concentrations in both PREOP and POD2.

^2^Those metabolites with an opposite fold change directionality at each time point.

^3^The 11-metabolite predictors used for the SVM prediction model at PREOP. 2-Oxobutanate is the only one found to be significant at both PREOP and POD2.

^4^The selected reaction monitoring (SRM) transition for this entry allows for detecting both itaconic acid and citraconic acid and is not specific to either. Metabolites included in this entry do not chemically behave as expected under the individual itaconic acid SRM, or the itaconic acid entry would have captured them.

Applying one predictive and one orthogonal component, the OPLS-DA model produced moderate separation between PREOP DEL and CNT, and minimal within group variation (Fig. 2c). Despite the good separation achieved in Fig. 2c, and high total sum of variation explained by the model (R2Y(cum) = 0.719), the estimation of model predictive performance was modest (Q2 = 0.203). Variable importance in projection (VIP) scores, which reflect both the loading weights and variability of response, bolstered previous univariate findings (Table 2a; Fig. 2e). The OPLS-DA model was initially validated using permutation testing and further tested for robustness by CV-ANOVA. The *p* values for permutation testing were pR2Y < 0.159 and pQ2 < 0.001, and for CV-ANOVA was < 2.4E−4, thus showing a statistically significant validation of the separation achieved between PREOP DEL and CNT when using this model. There were 11 metabolites with a VIP score > 2.0 (VIP > 1.0 is considered significant in distinguishing between classes), which included three of the four metabolites that showed a binomial test BH-corrected *p* value < 0.05. A support vector machine (SVM) prediction model^40^ using these 11 metabolites as predictors yielded an area under the curve (AUC) of 83.80% for the associated receiver operating characteristic (ROC) curve (Supplementary Fig. S2).

### Metabolites altered in delirium group at POD2

Fifty-three metabolites were found to have had significantly different concentrations between DEL and CNT at POD2 with a BH *p* value < 0.05 in at least two tests (Fig. 2b; Table 2b; Supplementary Table S7). Six of these metabolites were also altered at PREOP with three of them reversing the FC directionality. PREOP and POD2 associated metabolites represent candidate risk and disease markers, respectively. Therefore, the metabolites and/or their FC sign may be different because they represent different underlying biological mechanisms. Based on the 10,000 tenfold cross validation split datasets, all of the metabolites had *p* < 0.05 in at least two tests in all of the cases, and 28 of the metabolites had BH-corrected *p* < 0.05 in at least two tests in more than 50% of the cases (Supplementary Table S6).

Using one predictive and two orthogonal components, the OPLS-DA model produced clear separation between POD2 DEL and CNT with R2Y(cum) = 0.848 and predictive performance with R2Q = 0.344 (Fig. 2d,f). All VIP scores were at or above 1.0, supporting univariate findings (Table 2b; Fig. 2). The model was validated using permutation testing and evaluated for robustness with CV-ANOVA. The *p* values for permutation testing were pR2Y < 0.004 and pQ2 < 0.001, and for CV-ANOVA it was < 9.0E−7. These results support a statistically significant separation achieved between POD2 DEL and CNT when using this model. Based on both the statistical analysis and predictive modeling, metabolites at POD2 show significantly better discriminative power than at PREOP.

### Pathway enrichment analysis

**Figure 3:**
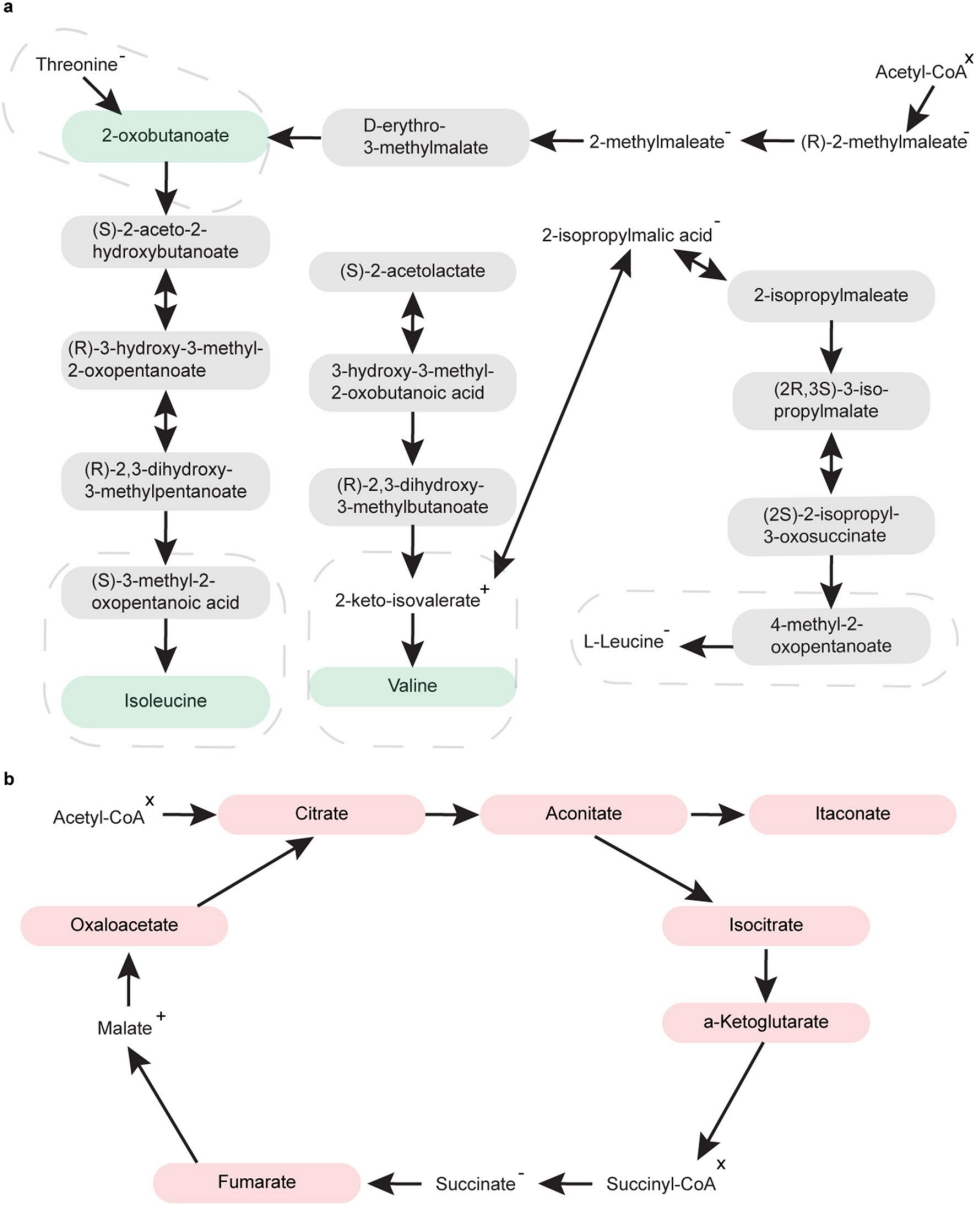
Pathway analysis of metabolites with significant differentially quantified concentrations using MetaboAnalyst^81–83^. Red indicates upregulation in DEL and green denotes downregulation. Grey represents metabolites not included in our targeted metabolomics protocol. Superscripts signify direction of change for the metabolites that did not exhibit significant differential concentrations. The +, −, and × superscripts indicate upregulated (in DEL), downregulated (in DEL), and signal too low or not present, respectively. (**a**) At PREOP, the valine, leucine, and isoleucine biosynthesis pathway was the only one with metabolites with significantly differentially quantified concentrations (FDR < 0.04). Dashed boxes represent the reactions that are specific to human. (**b**) At POD2, the citrate cycle pathway was the most significantly enriched.

At PREOP, the valine, leucine, and isoleucine biosynthesis pathway was the most significantly enriched (FDR < 0.04) (Table 3a; Supplementary Table S8; Supplementary Fig. S3). This canonical pathway is found in many species but the metabolic reactions specific to humans are noted by dashed boxes in Fig. 3a. These four reactions require the enzyme serine dehydratase or branched chain amino acid aminotransferase^41–43^. These chemical processes include eight metabolites, and of these, three of them were found to have significant differential concentrations (nominal *p* value < 0.05) at PREOP: 2-oxobutanoate, isoleucine, and valine. All three metabolites were downregulated in DEL (Fig. 3a).

**Table 3:** Biochemical pathways enriched with significant metabolites at preoperative (PREOP) and postoperative day 2 (POD2).

**a** Preoperative (PREOP)	Total	Expected	Hits	Raw p	FDR
Valine, leucine, and isoleucine biosynthesis	8	0.18	3	0.0005	0.04
Nicotinate and nicotinamide metabolism	15	0.33	3	0.0037	0.15
Pyrimidine metabolism	39	0.86	4	0.0092	0.26
One carbon pool by folate	9	0.20	2	0.0153	0.32
Glycine, serine, and threonine metabolism	33	0.72	3	0.0336	0.50
Arginine biosynthesis	14	0.31	2	0.0360	0.50

**b** Postoperative Day 2 (POD2)	Total	Expected	Hits	Raw p	FDR
Citrate cycle (TCA cycle)	20	0.65	6	2.30E−05	0.0019
Cysteine and methionine metabolism	50	1.06	6	4.70E−04	0.014
Pentose phosphate pathway	56	0.71	5	4.96E−04	0.014
Glyoxylate and dicarboxylate metabolism	32	1.03	5	0.003	0.062
Pyrimidine metabolism	39	1.26	5	0.007	0.120
Alanine, aspartate, and glutamate metabolism	28	0.90	4	0.011	0.155
Ascorbate and aldarate metabolism	8	0.26	2	0.025	0.265
Valine, leucine, and isoleucine biosynthesis	8	0.26	2	0.025	0.265
Pyruvate metabolism	22	0.71	3	0.032	0.295

Enriched pathways (*p* < 0.05) were identified using MetaboAnalyst 4.0^81–83^, which considers both metabolite over representation within a pathway and location importance^41,84^. A metabolite was included in biological systems analysis if it showed significant differentially quantified concentrations (nominal *p* value < 0.05 (PREOP), Benjamini–Hochberg (BH) *p* value < 0.05 (POD2)) in two or more statistical tests. “Total” represents the number of metabolites in the pathway, while “Hits” is the number of “input” metabolites found in the pathway.

At POD2, three pathways were found to be significantly enriched with our significant metabolites (N = 53) (Table 3b, Supplementary Table S8, Supplementary Fig. S3). The citrate cycle was the most significant pathway (FDR < 0.0019). This pathway included six significant metabolites which were all upregulated in DEL at POD2 (Fig. 3b). Furthermore, in Fig. 3b we show one additional metabolite, itaconate/itaconic acid, which is increased in delirium cases, but is not part of the classical citrate cycle. Itaconic acid is directly linked to the metabolite aconitate in this pathway.

## Discussion

In this paper, we applied targeted metabolomics to study delirium both pre- and postoperatively using a nested matched case-control design that maximizes efficiency and control of confounding factors. We identified metabolites associated with delirium both at PREOP and POD2 suggesting potential risk and disease markers. Our 11-metabolite predictive signature at PREOP, which achieved an AUC close to 84%, can be combined with other biomarker panels in a multi-omics approach. Systems biology analysis suggests that the ‘valine, leucine, and isoleucine biosynthesis’ and ‘citrate cycle’ pathways may be dysregulated in delirium. These altered pathways coincide with alternative energy pathways previously implicated in delirium pathogenesis in several studies^15,31,44^. Our findings suggest that alterations in primary and secondary energy production, amino acid synthesis metabolic pathways, and increased oxidative stress may be involved in the etiology and pathophysiology of delirium.

Metabolomics provides new insights into metabolic phenotypes and dysregulations of metabolic pathways underlying human disease and adds an important avenue for biomarker and therapeutic target discovery. However, experimental and computational challenges such as sample size, batch effect, peak drift, and data normalization hamper wide-spread use^45^. We established a robust workflow by using internal standards and pooled QC samples that provided reliable data for downstream analysis (RSD < 10% for > 98% of the metabolites in pooled QC samples).

Our study is one of only a few metabolomics studies of delirium. Pan et al. characterized the preoperative cerebrospinal fluid (CSF) metabolome of older patients (≥ 65 years) who developed delirium following elective hip or knee arthroplasty. They found that spermidine, putrescine, and glutamine were significantly upregulated in delirium patients compared to controls^30^. In a blood-based metabolomics study of delirium, Guo et al. took an untargeted metabolomics approach in characterizing preoperative serum metabolites of older surgical patients (between 65 and 80 years) undergoing hemiarthroplasty for hip fractures. They identified four metabolites associated with a higher risk of postoperative delirium (S-methylcysteine, linolenic acid, eicosapentaenoic acid, and linoleic acid) with alterations in energy metabolism and oxidative stress pathways^29^. In a follow-up study, Guo et al. profiled both PREOP and POD1 samples and reported imbalances between branched-chain and aromatic amino acids with perturbations in tricarboxylic cycle and oxidative stress pathways^31^. Although the specific metabolites and direction of dysregulation were not always consistent within the three studies (Supplementary Table S9), there were agreements in perturbed pathways.

There were six metabolites found to be significant at both time points; three displayed opposite directions in fold change noted in Table 2. This observation could be because the significant metabolites in the two timepoints imply different clinical/biological mechanisms (risk vs. disease factor). One of these, 2-oxobutanoate, is part of the 11-metabolite predictor used for the SVM prediction model at PREOP. Enrichment analysis using the 11-metabolite predictor revealed that 5 metabolites (N-acetyl-l-alanine, nicotinamide, Creatine, 2-oxobutanoate, Creatinine) are involved in metabolism of amino acids as well as 2 metabolites (Uracil, Uridine) are linked to pyrimidine ribonucleosides degradation. Uracil was the only metabolite linked to delirium by Guo et al.^31^. Disruption in amino acid metabolism has been linked to Alzheimer’s disease and dementia^46,47^. Another one of these metabolites, N-acetyl-glutamate (NAG), is a crucial enzymatic cofactor for the first step in the urea cycle, essential for liver ureagenesis and reduced levels are associated with Alzheimer’s disease^48^. (Supplementary Table S10).

Our systems biology approach indicates possible downregulation of the valine, leucine, isoleucine biosynthesis pathway at PREOP, for those patients who would go on to develop delirium following surgery. Valine, leucine, and isoleucine are essential branched-chain amino acids (BCAA). Essential amino acids are not naturally synthesized in the body and are obtained through diet. BCAAs contribute to energy production, and are key precursors in glutamate synthesis^49^. They play critical roles in the biochemistry of the brain and are important building blocks in proteinogenesis. The availability of these free form amino acids is a rate-limiting step in proteinogenesis. BCAAs are a subset of the large neutral amino acid (LNAA) class, which also includes the aromatic amino acids (AAA), tyrosine, tryptophan, and phenylalanine. LNAAs are key precursors for neurotransmitters that have been implicated in delirium, including glutamate, serotonin, dopamine, and norepinephrine^13,14^. BCAAs and AAAs compete for access to the central nervous system (CNS) through the large neutral amino acid transporter 1 (LAT1)^50–52^. With the absence of BCAAs, there is greater opportunity for the AAAs to reach CNS and generate their associated neurotransmitters: serotonin, dopamine, and norepinephrine.

We identified distinct metabolites and metabolic pathophysiological pathways linked to delirium development at POD2. Most significantly, potential upregulation in the citrate cycle pathway in delirium cases was predicted. This is a key pathway for energy metabolism. Six metabolites within the citrate cycle (oxaloacetate, fumarate, citrate, aconitate, isocitrate, α-ketoglutarate) were all increased in delirium cases, as was itaconic acid, which is generated by decarboxylation of aconitate. Interestingly, both plasma and CSF metabolomics comparison of patients with Alzheimer’s disease (AD) to matched cognitive healthy controls identified a similar increase of several citrate cycle intermediates such as citrate, aconitate, and α-ketoglutarate in AD^53,54^. Importantly, there is a well characterized reduction in activity of the rate limiting citrate cycle enzyme α-ketoglutarate dehydrogenase which converts α-ketoglutarate to succinyl-CoA^55,56^ that may be a key event in AD pathogenesis as well as various other neurodegenerative diseases due to oxidative stress^54^. Reduced  α-ketoglutarate dehydrogenase may result in accumulation of α-ketoglutarate. A disruption of energy homeostasis and increased oxidative stress because of increased citrate cycle activity may be a crucial event during delirium manifestation.

Glucose is the main fuel for the brain, but under glucose-depleted conditions like strenuous exercise, fasting, or starvation; the ketone bodies, β-hydroxybutyrate (βOHB) and acetoacetate (AcAc), are the main alternative fuel sources. These small lipid-derived molecules are converted to acetyl-CoA and then oxidized in the citrate cycle to produce adenosine triphosphate and carbon dioxide^57^. Kealy and colleagues showed that energy metabolism dysregulation was sufficiently capable of triggering an acute cognitive dysfunction in a delirium mouse model^44^. Under normal conditions in humans, these ketones are present in plasma in an approximate 2:1 βOHB:AcAc ratio^58^. At PREOP, the delirium cohort displayed a decrease in βOHB (FC = − 1.69, nominal *p* value = 0.01), and then an upregulation of AcAc (FC = 1.54, BH *p* value = 0.00075) at POD2, elucidating an alteration in energy metabolism. At POD2 our participants were in a fasting state, which supports an increase in acetoacetate, but this upregulation should be uniform across both phenotypes. This disparity points to potential energy pathway alteration in the delirium group that is not observed in the control group. These findings support previous hypotheses that energy metabolism dysregulation is a possible driver in delirium pathophysiology^29,31,44^.

While most of the metabolites identified in this study have not been previously linked to delirium risk or development, a few of these metabolites have been previously linked to inflammation, cognitive function, or neurological diseases. Most prominent is kynurenic acid, which our data show to be significantly increased in delirium cases at POD2. Kynurenic acid, a degradation product of l-tryptophan, is a neuromodulator interacting with NMDA, nicotinic, and GPR35 receptors. It plays a role in various neurophysiological functions as well as neurological diseases and inflammatory processes. Overexpression of kynurenic acid in several neurological diseases is associated with confusion^58^, while reduction of kynurenic acid in mice improves cognitive function^59,60^. Both kynurenine and tryptophan are differentially quantified concentrations at POD2 in delirium samples at nominal, not BH-corrected, *p* value (< 0.05 in all three statistical tests) with kynurenine, like kynurenic acid, being increased and tryptophan being decreased in delirium samples. Thus, kynurenic acid may be one key metabolite associated with delirium development.

Despite our strengths in experimental design, established cohort, developed workflow, and data analysis methods, several limitations of this study are of note. The statistical power at PREOP was still insufficient to yield metabolites that passed two or more BH-corrected statistical tests implying the need for more samples. Second, the SAGES study was conducted within a single geographic region, limiting the generalizability of our results across other populations. Third, since only plasma samples were analyzed, it is difficult to assess whether similar metabolic changes occur in the brain and have functional and causal consequences with regard to delirium risk or development. Therefore, the identification of disruptions in several metabolic pathways and the link to delirium pathogenesis and pathophysiology are putative and need to be further validated. Fourth, since delirium had already occurred in most of our participants at the time of the POD2 blood draw, we are unable to completely disentangle metabolic changes that are on the causal pathway to delirium from those that result from delirium. Finally, targeted metabolomics is restricted by detection of the predefined metabolites established by the specific protocol that may result in the omission of metabolites involved in delirium.

In conclusion, we have identified metabolites associated with delirium at both pre- and post-operative time points that may enhance our understanding of the etiology and pathophysiology of delirium. Our findings highlight potential dysregulation in energy production pathways at both PREOP and POD2 paving the way for future studies, which should expand upon these findings through exploration of key enzymes involved in these pathways. The metabolomic findings at PREOP have the potential to be combined with other biomarkers to develop a predictive signature, which can be used to target personalized, preventative interventions to reduce delirium incidence. Additionally, our analysis of the metabolome at POD2 could improve our understanding of the disease mechanism, which may lead to better strategies to ameliorate the delirium process. Overall, the established workflow and data analysis provide a two-faceted approach to delirium metabolomics providing a comprehensive assessment of the biochemical activity associated with this syndrome.

## Methods

### Study participants

The Successful Aging after Elective Surgery (SAGES) study is a prospective observational study of older adults undergoing major elective non-cardiac surgery and was designed to understand novel risk factors and long-term outcomes of delirium^59,60^. SAGES enrolled patients ≥ 70 years old scheduled for major noncardiac surgery, including total knee or hip replacement, cervical or lumbar laminectomy, abdominal aortic aneurysm repair, lower extremity vascular bypass, or colectomy. Patients received either general or spinal anesthesia. Major inclusion and exclusion criteria have been published^59,60^. Patients underwent a detailed screening process to exclude dementia based on medical record review, capacity assessment, patient or family report of dementia diagnosis, and cognitive testing using the Modified Mini-Mental State Examination^61^. In addition, patients underwent a neurocognitive battery at baseline, which was used to compute the general cognitive performance (GCP) summary measure^62^. Comorbidities were identified from medical record review by trained physician abstractors and scored based on the Charlson Comorbidity Index.

Informed consent for study participation was obtained from all subjects according to procedures approved by the institutional review boards of Beth Israel Deaconess Medical Center (BIDMC) and Brigham and Women’s Hospital, the two surgical sites, and Hebrew SeniorLife, the study coordinating center, all located in Boston, Massachusetts. All experiments, methods, and analyses conducted in the current manuscript were performed in accordance with relevant guidelines and regulations of the institutional review board (IRB) of all participating institutions.

### Data collection

Postoperative delirium was determined daily throughout hospitalization, supplemented with a validated chart review to identify cases that may have been missed during daily interviews. Delirium was assessed using the Confusion Assessment Method (CAM) diagnostic algorithm^63,64^. The presence of delirium by chart review was adjudicated by at least two delirium experts, and discordance was resolved through consensus^65^. Patients were considered delirious if delirium was present on either the CAM or the chart review method on any postoperative day; otherwise, patients were considered non-delirious. If delirium was absent during the entire hospitalization, subsyndromal delirium was defined as (i) an acute change in mental status or fluctuation, (ii) at least one CAM core feature (inattention, disorganized thinking, altered level of consciousness), and (iii) at least one other CAM supporting feature (disorientation, perceptual disturbance, delusion, psychomotor agitation, psychomotor retardation, or inappropriate behavior)^66^.

### Creation of nested matched case-control sample

From the entire SAGES cohort, a matched delirium/no-delirium sample was previously identified to examine inflammatory markers and plasma proteomics^67,68^. Delirium cases (DEL) were defined as patients with delirium on POD2. No delirium controls (CNT) were defined as patients with no delirium or no subsyndromal delirium during their hospital stay. DEL/CNT pairs were matched on six variables (age and baseline GCP within five years, and an exact match for sex, presence of vascular comorbidity, surgery type, and Apolipoprotein E [APOE] ε4 status) (Table 1). Vascular comorbidity was present if the participant had at least one Charlson diagnosis related to vascular disease: myocardial infarction, congestive heart failure, peripheral vascular disease, cerebrovascular disease, hemiplegia, and diabetes or diabetes with end organ damage. For APOE genotype, DNA was extracted from cellular material in the blood and genotyped at the Partners Center for Personalized Medicine. Patients with at least one ɛ4 allele were defined as APOE ɛ4 carriers. To limit heterogeneity of surgical procedure only patients who underwent an orthopedic procedure were included in this manuscript 66 matched pairs. Fourteen pairs with low plasma volume in the biorepository were excluded, yielding n = 52 matched pairs (104 participants) for inclusion in this study.

### Blood collection and processing for plasma

Heparinized plasma samples were collected and stored as part of the SAGES study^59^ as previously described^68^. PREOP and POD2 timepoint plasma was utilized in the present study. For all of the samples, blood was collected in a non-fasting state at PREOP and in a fasting state at POD2. A pooled control sample was created from twenty PREOP samples (ten males and ten females) from patients de-enrolled from the SAGES study. Metabolites were extracted from equal volumes of heparinized plasma (100 µl) by ice-cold methanol precipitation added to a final concentration of 80% (vol/vol).

### Targeted metabolomics

Equal volumes per sample of methanol extracted metabolites were run on a 5500 QTRAP (SCIEX, Framingham, MA) mass spectrometer using a previously published targeted methodology developed by the BIDMC Mass Spectrometry Core^69^. This protocol utilizes a targeted selected reaction monitoring (SRM), positive/negative ion-switching, mass spectrometry-based metabolomics platform suitable for bodily fluids, cells, and fresh and fixed tissue^69^.

### Description of data

To accommodate for the maximum sample loading capacity of the 5500 QTRAP 60 samples, six unique sample preps and mass spectrometry runs were performed for a total of 52 matched DEL/CNT pairs at PREOP and POD2 that resulted in analysis of a total of 208 samples. Each experimental run—except for the final one, which was abridged due to sample limitations—included ten matched pairs, three internal spike-in standards, thirteen pooled quality control plasma samples (QC), three conditioning samples and four blanks (Fig. 1, Supplementary Fig. S1). In the supplementary material we explain the sample order for each run, in detail.

### Preprocessing of metabolomics data

Overall, 315 metabolites were measured using previously published targeted methodology (Supplementary Table S1)^69^. A metabolite was considered “present” if it was measured in at least 50% of the samples within a phenotypic group (PREOP CNT, PREOP DEL, POD2 CNT, POD2 DEL).

Signal drift was corrected with statTarget using a quality control-based machine learning algorithm: random forest signal correction (QC-RFSC)^70,71^. This allowed for the integration of the six different experimental runs using the pooled QC samples. Metabolites deemed absent were omitted while signal imputation was performed on the remaining using the k-nearest neighbor (knn) method^72^.

NormalizeMets R package was used to normalize the metabolomics data matrix to previously selected internal standards using the NOMIS: normalization using optimal selection of multiple internal standards technique^71,73,74^.

### Statistical analysis

Differential metabolite concentration was assessed using both parametric and nonparametric statistical tests to account for the degree, direction, and rank of difference between DEL and CNT groups at both PREOP and POD2 time points. For all statistical tests (paired t-test, binomial test, and Wilcoxon signed-rank test), the Benjamini–Hochberg (BH) procedure was applied to correct for multiple hypotheses testing^75^. These results were further tested for robustness using tenfold split analysis at a 5% significance level, with 80% power, and run 10,000 times (Supplementary Table S6). If a metabolite came up as significant in the majority of the splits (≥ 50%), this conferred a level of confidence in the observed differential concentration. Fold-change (FC) of metabolite concentration was calculated by applying the one-step Tukey’s biweight algorithm on FC values (DEL/CNT) for each paired sample^76^. This provides a robust estimation of the FC for each individual metabolite that is unaffected by outliers. The FCs of the metabolites that are downregulated in the delirium group are indicated using the negative sign, e.g., a FC of − 2 implies two-fold downregulation in the delirium group.

Multivariate statistical analysis was performed using the ropls package in the statistical program R^71,77^. Potential outliers were identified using principal component analysis (PCA) and Mahalanobis distance^78^ and were omitted prior to the application of orthogonal partial least squares-discriminant analysis (OPLS-DA), a supervised multivariate separation method^77,79^. The model was validated through sevenfold cross-validation, cross validation-analysis of variance (CV-ANOVA)^80^, and response permutation testing (n = 1000) using the fit metrics R2X, R2Y, and Q2.

### Systems biology analysis

Systems biology analyses of delirium-related metabolites were performed using MetaboAnalyst 4.0 (Montreal, Quebec, Canada)^81–83^, which takes a bifurcated analytic approach that includes metabolite over-representation and pathway topology^41,84^. A metabolite was included in biological systems analysis if it showed significant differential concentration (nominal *p* value < 0.05 (PREOP), BH *p* value < 0.05 (POD2)) in two or more statistical tests. At PREOP, nominal *p* value was used in lieu of BH *p* value because systems analysis requires more metabolites than those that met the BH significance cut-off < 0.05.

Hypergeometric testing was used to assess if delirium-related metabolites were overrepresented in a pathway more than expected by chance^81,82^. A pathway was considered enriched if the false discovery rate (FDR) adjusted *p* value was < 0.05. In topology analysis, perturbed metabolites located in critical or central positions are deemed to have a greater impact on a pathway.

## Supplementary Information

Supplementary Tables.

Supplementary Figures.
