# Supplementary material for: Targeted metabolomics analysis of postoperative delirium
Source: Sci Rep. 2021 Jan 15;11:1521. doi: 10.1038/s41598-020-80412-z (PMC7810737; doi:10.1038/s41598-020-80412-z)
Supplement: Supplementary file 2 — Supplementary Figures. [file 41598_2020_80412_MOESM2_ESM.pdf]

Targeted Metabolomics Analysis of Postoperative Delirium

Bridget A Tripp[1,2*], Simon T Dillon[3,4,6], Min Yuan[5], John M Asara[4,5], Sarinnapha M Vasunilashorn[4,6,7], Tamara G Fong[4,8,9], Eran D Metzger[10,11], Sharon K Inouye[4,6,9], Zhongcong Xie[4,12], Long H Ngo[4,6,7], Edward R Marcantonio[4,6£], Towia A Libermann[3,4,6£], Hasan H Otu[1£]

[1]Department of Electrical and Computer Engineering, University of Nebraska-Lincoln, [2]PhD Program of Complex Biosystems, University of Nebraska-Lincoln, [3]Genomics, Proteomics, Bioinformatics and Systems Biology Center, Beth Israel Deaconess Medical Center, [4]Harvard Medical School, [5]Division of Signal Transduction and Mass Spectrometry Core, Beth Israel Deaconess Medical Center, [6]Department of Medicine, Beth Israel Deaconess Medical Center, [7]Harvard T.H. Chan School of Public Health, [8]Department of Neurology, Beth Israel Deaconess Medical Center, [9]Aging Brain Center, Marcus Institute for Aging Research, Hebrew SeniorLife, [10]Department of Medicine, Hebrew SeniorLife, [11]Department of Psychiatry, Beth Israel Deaconess Medical Center, [12]Department of Anesthesia, Critical Care and Pain Medicine, Massachusetts General Hospital

[£]Co-senior authors

*Corresponding Authors

Send correspondence to Bridget A. Tripp, Department of Electrical and Computer Engineering University of Nebraska-Lincoln, Nebraska Hall E419. P.O. Box 880511, Lincoln, NE 68588, Tel: (402) 472-3771, Fax: (402) 472-4732, Email: bridget.tripp@huskers.unl.edu

Figure S1: Run sample order: The injection order and sample type for each analytical run

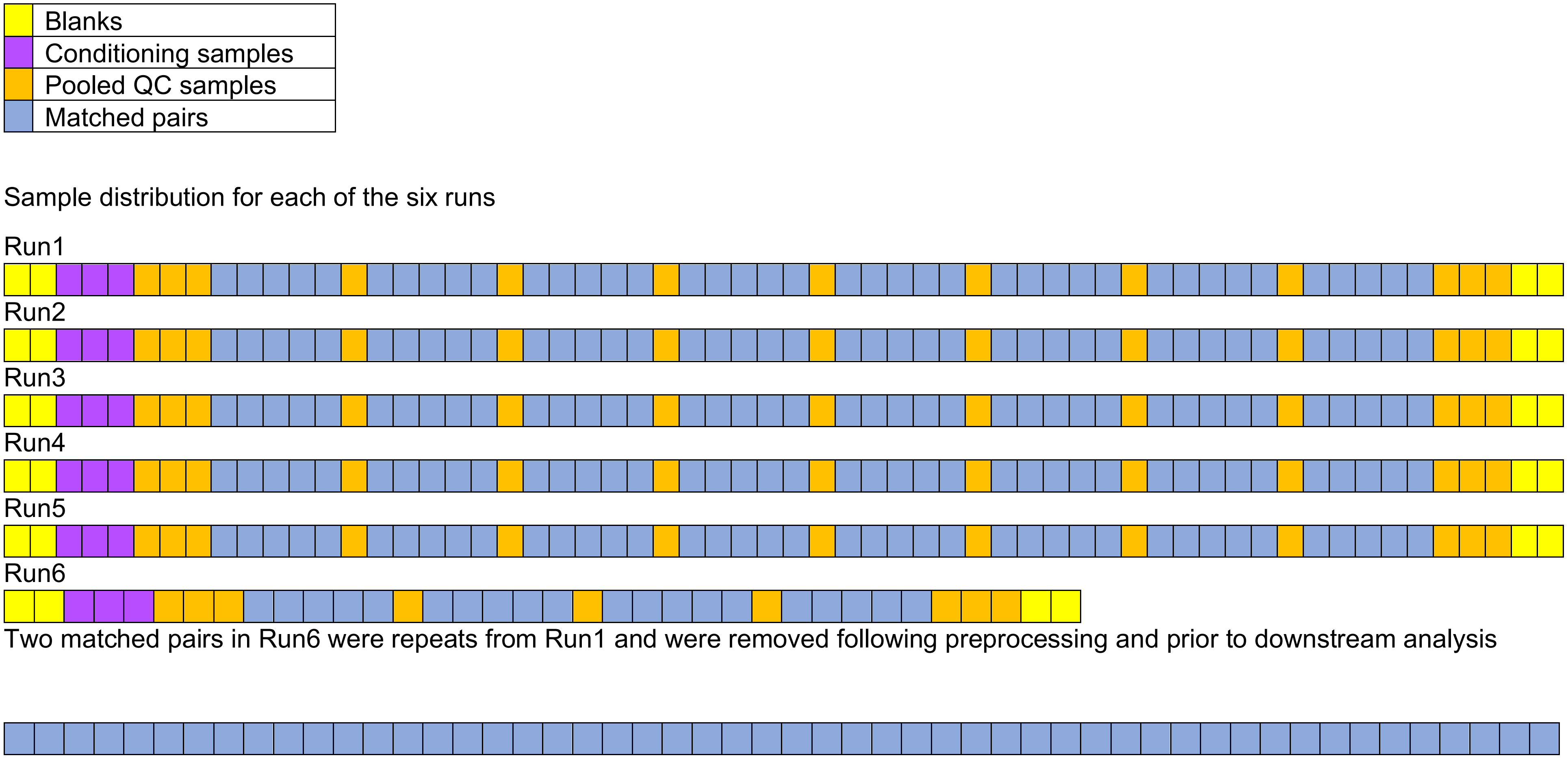

52 matched pairs for 208 samples

The injection order was specifically designed to combine multiple analytical runs while controlling for peak drift and internal and external noise. The blank and conditioning samples were used to minimize carryover from previous samples and identify background noise. The pooled quality control (QC) samples were used to correct for batch and peak drift.

Figure S2: ROC[1] curve for SVM[2] prediction model using 11-metabolite predictors at PREOP[3]

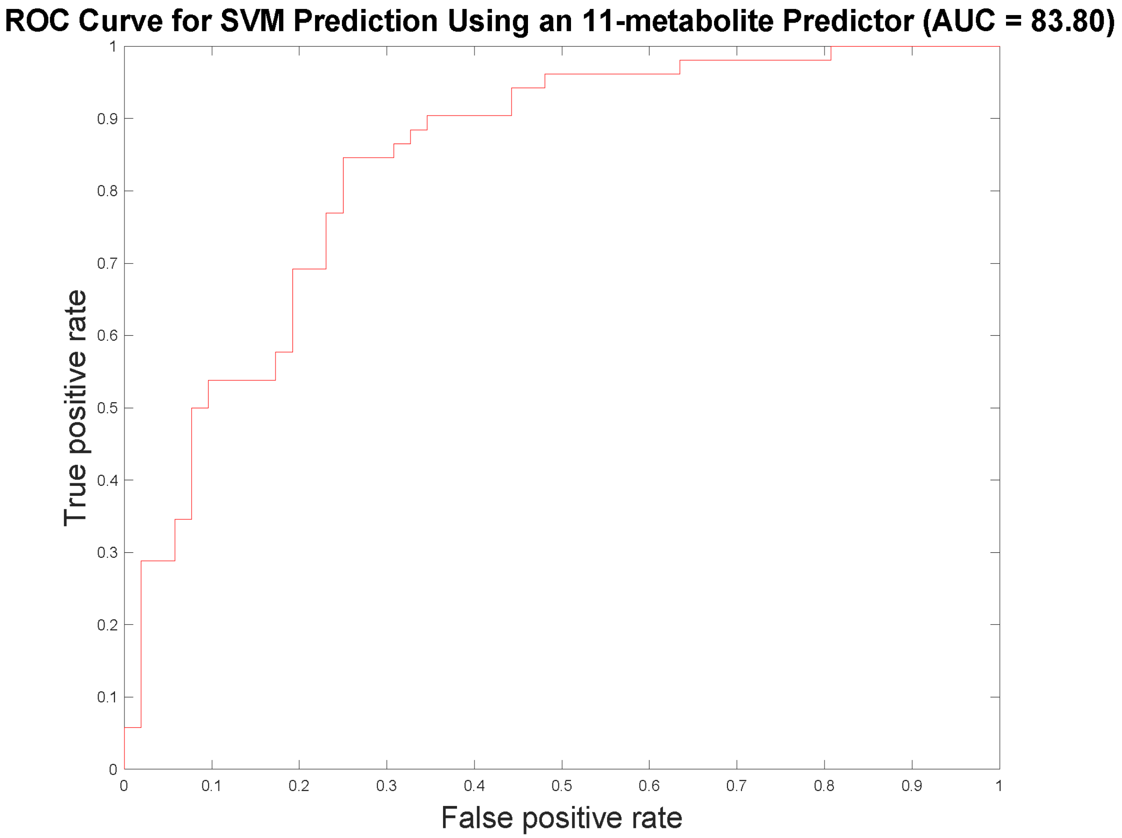

[1]Receiver operating characteristic, [2]support vector machine, [3]Preoperative

The eleven predictor metabolites used included: uracil, proline, 3-hydroxybuterate, trehalose-6-phosphate, N-acetyl-L-alanine, creatine, uridine, 2-oxobutanoate, N-acetyl-glutamate, creatinine, nicotinamide.

Figure S3: Significant Pathways using MetaboAnalyst and KEGG pathways [1-6]

a Valine, Leucine, Isoleucine Biosynthesis

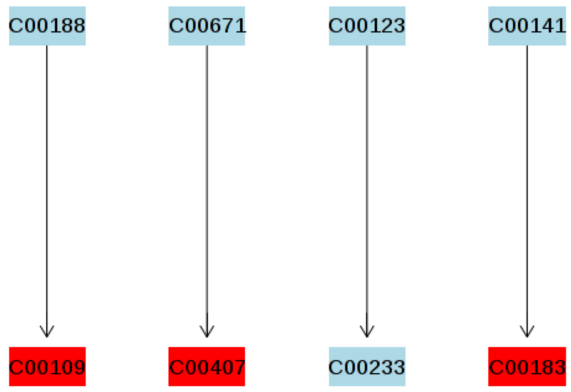

b Nicontinate and Nicotinamide Metabolism

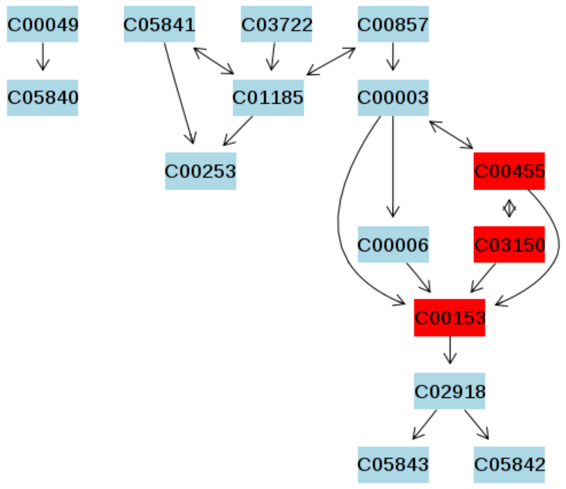

c Pyrimidine Metabolism

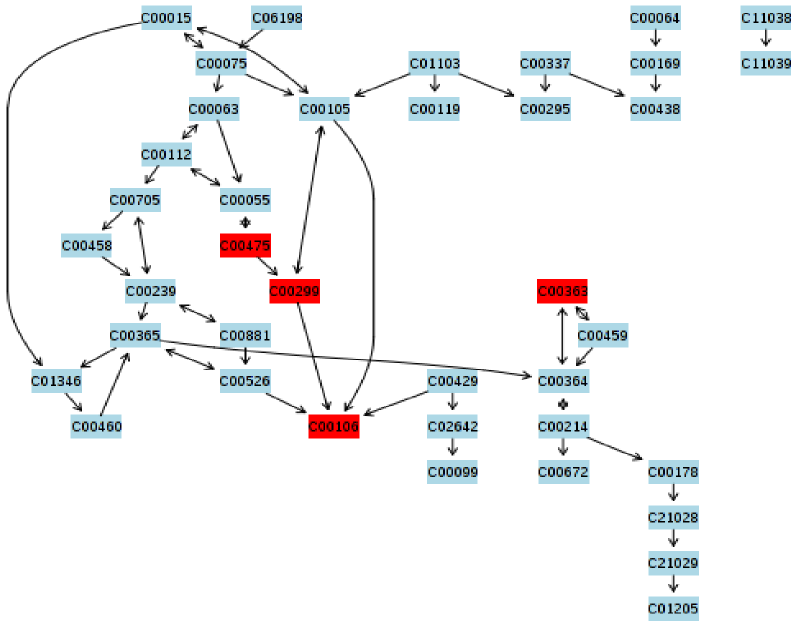

d Citrate Cycle (TCA cycle)

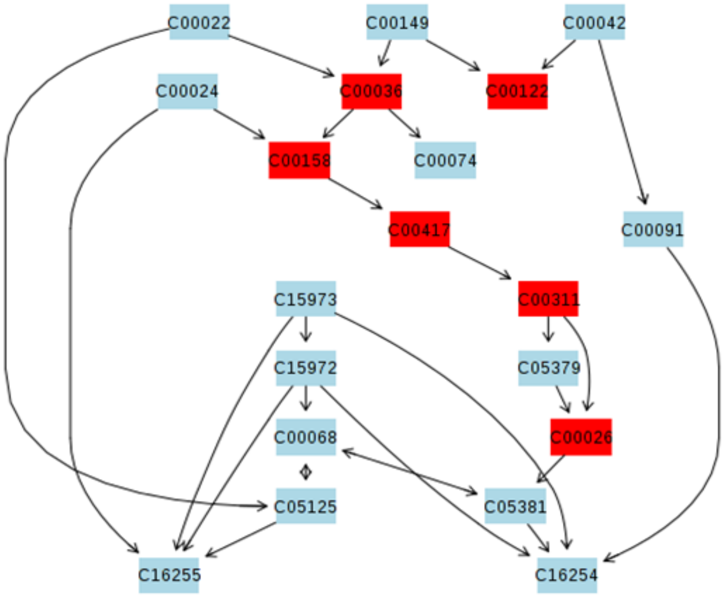

e Cysteine and Methionine Metabolism

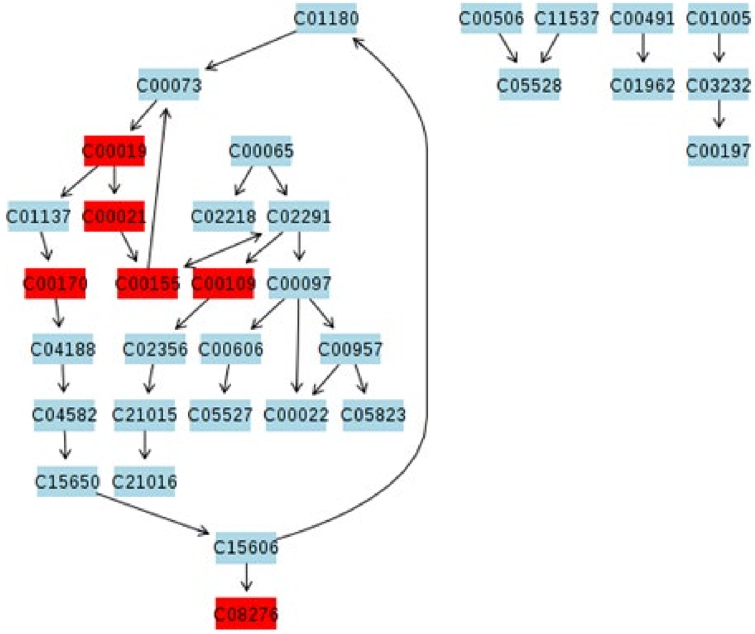

f Pentose Phosphate Pathway

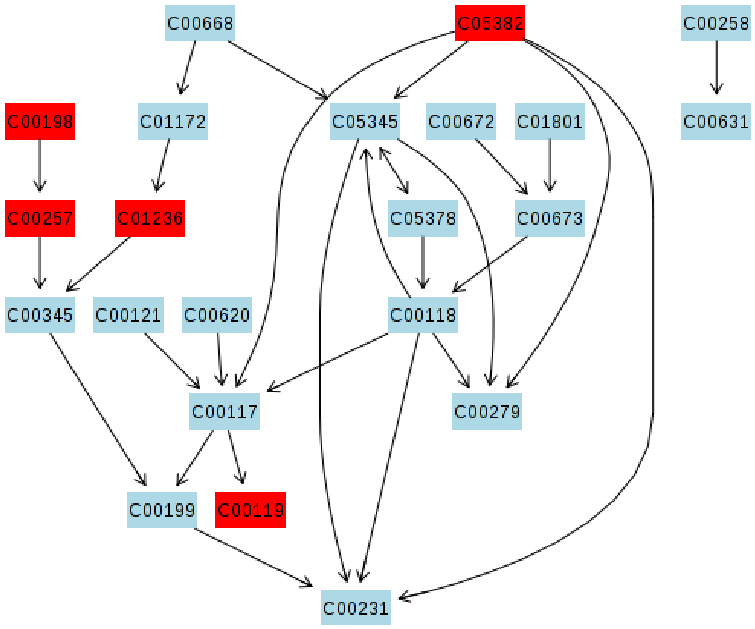

Red denotes those metabolites found to be significant between delirium and control groups. Top three pathways enriched with significant metabolites at PREOP **(a, b, c)** and POD2 **(d, e, f)**.

References

1. Xia J, Sinelnikov IV, Han B, Wishart DS. MetaboAnalyst 3.0--making metabolomics more meaningful. *Nucleic Acids Res* 2015; **43**(W1)**:** W251-257.

2. Xia J, Wishart DS. Using MetaboAnalyst 3.0 for comprehensive metabolomics data analysis. *Curr Protoc Bioinformatics* 2016; **55:** 14.10.11-14.10.91.

3. Chong J, Wishart DS, Xia J. Using MetaboAnalyst 4.0 for comprehensive and integrative metabolomics data analysis. *Current Protocols in Bioinformatics* 2019; **68**(1)**:** e86.

4. Kanehisa M, Goto S. KEGG: kyoto encyclopedia of genes and genomes. *Nucleic Acids Res* 2000; **28**(1)**:** 27-30.

5. Kanehisa M, Furumichi M, Tanabe M, Sato Y, Morishima K. KEGG: new perspectives on genomes, pathways, diseases and drugs. *Nucleic Acids Res* 2017; **45**(D1)**:** D353-D361.

6. Kanehisa M, Sato Y, Furumichi M, Morishima K, Tanabe M. New approach for understanding genome variations in KEGG. *Nucleic Acids Res* 2019; **47**(D1)**:** D590-d595.
